# Clinical Value of Tocilizumab in Reducing Mortality in Refractory Septic Shock in Children with Hematologic and Non-Hematologic Diseases

**DOI:** 10.3390/cells14060441

**Published:** 2025-03-16

**Authors:** En-Pei Lee, Jainn-Jim Lin, Shih-Hsiang Chen, Oi-Wa Chan, Ya-Ting Su, Man-Ru Hsiao, Shao-Hsuan Hsia, Han-Ping Wu

**Affiliations:** 1 Division of Pediatric Critical Care Medicine, Department of Pediatrics, Chang Gung Memorial Hospital at Linko, Kweishan, Taoyuan 33305, Taiwan; pilichrislnp@gmail.com (E.-P.L.); lin0227@adm.cgmh.org.tw (J.-J.L.); oiwamail@gmail.com (O.-W.C.); 2 College of Medicine, Chang Gung University, Taoyuan 33305, Taiwan; samechen@cgmh.org.tw (S.-H.C.); nju83wu6@gmail.com (Y.-T.S.); lovefentanyl@cgmh.org.tw (M.-R.H.); 3 Divisions of Hematology and Oncology, Department of Pediatrics, Chang Gung Memorial Hospital, Chang Gung University, Taoyuan 33315, Taiwan; 4 Division of Pediatric Endocrinology and Genetics, Department of Pediatrics, Chang Gung Memorial Hospital, Taipei 10507, Taiwan; 5 Department of Pharmacy, Chang Gung Memorial Hospital, Taoyuan 33315, Taiwan; 6 Physician Assistant Department, University of Maryland Eastern Shore, Princess Anne, MD 21853, USA; 7 Department of Pediatrics, Chiayi Chang Gung Memorial Hospital, Chiayi 61363, Taiwan

**Keywords:** biomarkers, septic shock, cytokine storm, interleukin-6, children

## Abstract

Background: Pediatric sepsis remains the main cause of morbidity and mortality among children. Interleukin (IL)-6 is usually produced after infection, and elevated IL-6 levels may cause multisystemic damage. This study aimed to evaluate the effect of tocilizumab, an IL-6 receptor antibody, on children with septic shock. Methods: We conducted a retrospective cohort study of children diagnosed with septic shock and admitted to the pediatric intensive care unit (PICU) between 2018 and 2024. Tocilizumab was administered within 24 h to patients with high IL-6 levels who developed refractory septic shock. Outcomes, including 28-day mortality, morbidity, length of PICU stay, and shock duration, were analyzed between septic children with different etiologies and differed treatments. Results: Fifty-four children with refractory septic shock were included. Patients treated with tocilizumab (n = 21) showed improved outcomes compared to those without tocilizumab (n = 33), including shorter PICU stays and lower mortality rates (14.2% vs. 54.5%, *p* = 0.03). Subgroup analysis revealed that in the non-hematologic group, tocilizumab-treated patients had a 0% mortality rate compared to 50% in untreated patients (*p* = 0.006). In the hematologic group, tocilizumab-treated patients exhibited a 27.2% mortality rate compared to 61.5% in untreated patients (*p* = 0.09). Trends in IL-6 levels (D1 to D7) were significantly higher in non-survivors compared to survivors and in patients with hematological malignancies compared to those without. No adverse events, including secondary infections or long-term liver impairment, were observed. Conclusions: Tocilizumab appears to mitigate systemic inflammation and improve outcomes in children with refractory septic shock and elevated IL-6 levels. Further prospective studies are warranted to confirm these findings and establish treatment guidelines.

## 1. Introduction

Pediatric sepsis remains the main cause of morbidity and mortality among children and is an important public issue worldwide [1]. Pediatric septic shock accounts for more than 8% of all critically ill children and causes approximately 4.5 million deaths each year worldwide [1]. A previous study reported that approximately one-third of deaths in pediatric intensive care units (PICUs) are caused by pediatric sepsis [1]. Mortality rates for pediatric sepsis range from 4% to 50%, depending on disease severity and risk factors [2,3]. Most patients with pediatric sepsis die from “refractory septic shock” or multiple organ dysfunction, with mortality rates as high as 40–80% [1,4]. Many deaths occur within the first 72 h of treatment, highlighting the importance of early recognition and appropriate management [5,6,7].

Sepsis is defined as life-threatening organ dysfunction resulting from dysregulated host responses to infection [8]. Studies have demonstrated that enormous systemic inflammation and high cytokine levels are associated with poor prognosis [9,10]. Among the many cytokines, interleukin (IL)-6 is usually produced after infection, and elevated IL-6 levels may cause multisystemic damage, such as disseminated intravascular coagulation, endothelial dysfunction, and myocardial impairment [11]. One prospective meta-analysis reported that the administration of IL-6 antagonists to patients with coronavirus disease 2019 (COVID-19) was associated with lower all-cause mortality 28 days after randomization [12]. One preliminary experience in adults suggested that the use of the IL-6 receptor antibody tocilizumab is beneficial in patients with hematological malignancy who suffer from elevated IL-6 levels caused by severe sepsis [13]. However, research on IL-6 receptor antibodies for cytokine storms caused by sepsis in children is limited. Here, this study aimed to overvalue the effect of tocilizumab for pediatric patients with septic shock having excessive IL-6 levels.

## 2. Materials and Methods

### 2.1. Patient Population

This retrospective cohort study included pediatric patients diagnosed with septic shock and treated in the PICU at Chang Gung Children’s Hospital, Taiwan, between January 2018 and February 2024. The Institutional Review Board approved the study (IRB Number: 202300482B0). Clinical data, including demographic information, underlying disease, infection source, proven pathogens, Pediatric Risk of Mortality III (PRISM III) scores, laboratory parameters, vasoactive-inotropic scores (VIS), and patient outcomes, were collected.

### 2.2. Study Design

Pediatric septic shock was defined as cardiovascular dysfunction (hypotension, need for vasoactive agents) or impaired organ perfusion based on the 2020 Surviving Sepsis Campaign (SSC) [14]. Therapeutic strategies were based on the 2020 SCC [14]. Fluid resuscitation (40–60 mL/kg) was administered over the first hour if hypotension developed. Fluid-refractory septic shock was defined as persistent shock despite up to 60 mL/kg of fluid resuscitation. Vasoactive agents were administered if the patient continued to have evidence of abnormal perfusion in patients with fluid-refractory septic shock. In addition to antibiotic use, all patients with fluid-refractory septic shock received tocilizumab treatment. In our cohort, patients with fluid-refractory septic shock were divided into 2 groups based on time. From January 2022 to February 2024, tocilizumab was administered in children with elevated serum L-6 levels once the diagnosis of fluid-refractory septic shock was made. At another earlier time point, from January 2018 to December 2021, patients with fluid-refractory septic shock did not receive tocilizumab. Patient outcomes, including shock duration, 28-day mortality, and morbidity, were further collected and analyzed.

### 2.3. Serum IL-6 Levels Measurement

Serum IL-6 levels were measured at the time of refractory septic shock diagnosis and tracked on days 3, 5, and 7. The Elecsys IL-6 immunoassay is a diagnostic test that quantifies interleukin-6 in human serum and plasma using electrochemiluminescence on cobas e801 series analyzers (Hitachi., JAPAN). It has a measuring range of 1.5–50,000 pg/mL, a limit of quantitation of 2.5 pg/mL, and inter-assay precision of 17.4% at low levels and 2.0% at high levels, with a reference interval of less than 7 pg/mL.

### 2.4. Definition

The vasoactive-inotropic score (VIS) was defined as dopamine dose (mcg/kg/min) + dobutamine dose (mcg/kg/min) + 100 × epinephrine dose (mcg/kg/min) + 100 × norepinephrine dose (mcg/kg/min) 10 × milrinone dose (mcg/kg/min) + 10,000 × vasopressin dose (units/kg/min) [15].

## 3. Results

### 3.1. Patient Characteristics

Table 1 outlines the demographics and baseline characteristics of patients treated with and without tocilizumab. Among the study population, 33 patients were treated without tocilizumab during the earlier period (2018–2021), and 21 patients received tocilizumab during the later period (2022–2024). There were no significant differences in demographic variables such as age, sex, weight, underlying conditions, or infection site between the two groups. Similarly, disease severity and hemodynamic parameters, including the Pediatric Risk of Mortality III (PRISM III) score, vasoactive-inotropic scores (VIS), heart rate, and mean arterial pressure, were comparable between the groups. Bloodstream infections were the most common type of infection in both cohorts. All patients received broad-spectrum antibiotics effective against the pathogens. Notably, the outcomes were significantly better in the tocilizumab-treated group, with a shorter ICU stay and lower mortality rate (14.2% vs. 54.5%, *p* = 0.03).

### 3.2. Management and Outcome of Concomitant Tocilizumab Therapy

In the cohort, when refractory septic shock developed, in addition to broad-spectrum antibiotic usage and optimization of hemodynamics, the trend of IL-6, CRP, and procalcitonin levels were checked initially and every 2–3 days. All patients received a single dosage of tocilizumab (<30 kg: 12 mg/kg IV; >30 Kg: 8 mg/kg IV) after the development of refractory septic shock within 24 h [16]. All patients experienced a decrease in IL-6 levels after tocilizumab administration.

Table 2 compares patients without hematological malignancy who were treated with and without tocilizumab. There were no significant differences in demographic variables, disease severity, or hemodynamic parameters between the two groups. However, outcomes were significantly improved in patients treated with tocilizumab, including a shorter ICU stay and a lower mortality rate (0% vs. 50%, *p* = 0.006).

Table 3 presents the comparisons between patients with hematological malignancy treated with and without tocilizumab. Similar to the non-hematological malignancy group, demographic variables, disease severity, and hemodynamic parameters showed no significant differences between the two groups. Patients treated with tocilizumab demonstrated better outcomes, including a shorter ICU stay and a lower mortality rate (27.2% vs. 61.5%, *p* = 0.09).

**Figure 1:**
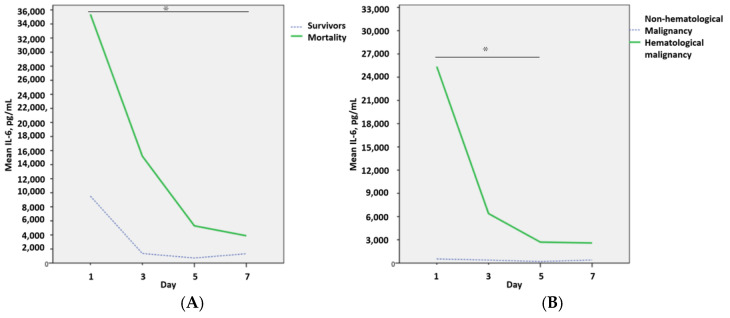
IL-6 trends in survivors vs. non-survivors (**A**). IL-6 trends in patients with and without hematological malignancies (**B**). * Statistical significance was set at *p* < 0.05.

The initial IL-6 was significantly higher in the non-survivors, and the trends of IL-6 (D1 to D7) were all significantly higher in the non-survivors compared with survivors (Figure 1A). Among patients treated with tocilizumab, the IL-6 trend (D1 to D7) was significantly hematological malignancy compared to those without hematological malignancy (Figure 1B).

Overall, patients treated with tocilizumab exhibited better outcomes in this cohort. When stratified into hematologic and non-hematologic groups, both groups receiving tocilizumab demonstrated improved outcomes compared to those who did not receive the treatment, including shorter ICU stays and lower mortality rates. Notably, none of the patients underwent hemodialysis due to acute kidney injury. Furthermore, no complications associated with tocilizumab, such as secondary infections or long-term liver impairment, were observed in any patients treated with the drug.

## 4. Discussion

This was the first pediatric study to analyze the use of tocilizumab as an adjuvant therapy for pediatric refractory septic shock. A preliminary study suggested that tocilizumab can help mitigate systemic inflammation and reduce the risk of multiorgan dysfunction with excessive IL-6 levels after the development of refractory septic shock.

One of the main pathophysiologies of sepsis is the upregulation of both pro- and anti-inflammatory pathways. Many proinflammatory cytokines, such as IL-1, IL-6, IL-8, IL-12, IL-18, TNF-a, and IFN-r, are highly expressed, leading to cytokine storms after sepsis develops [17]. Excessive cytokine levels result in progressive tissue damage, resulting in multiorgan impairment. A previous study reported that among the various cytokines, IL-6 was the most valuable cytokine associated with sepsis severity and outcome prediction [18]. Among patients with septic shock, survivors usually show a decreasing trend of IL-6 levels, and non-survivors usually show increasing IL-6 levels [18].

Molecular-targeted sepsis therapies for elevated cytokines have been studied for 40 years [17]. The use of neutralizing antibodies to block proinflammatory cytokines and reduce morbidity in sepsis was the main research field. TNF-A- and IL-1-neutralizing antibodies proved effective in animal models but did not have beneficial effects in humans with sepsis [19]. Studies analyzing proinflammatory cytokines, such as IL-8, IL-12, IL-17A, and IL-18, and anti-inflammatory cytokines, such as IL-4, IL-10, and IL-11, have reported no effective neutralizing antibodies to improve the outcomes of human sepsis [19].

In recent studies, the early use of the IL-6 receptor-neutralizing antibody tocilizumab has shown survival benefits in patients with COVID-19 infection and adult patients with hematological malignancies suffering from sepsis [12,13]. This study identified that tocilizumab may have a survival benefit in pediatric refractory septic shock. Kumar et al. reported that in adults with sepsis, IL-6 levels decreased significantly from day 1 to day 7 in survivors [20]. Although this study was not a randomized controlled trial, we also observed similar results in that all patients with refractory septic shock presented with high IL-6 levels initially, which then significantly decreased after receiving tocilizumab, improving the survival rate. During recent years in our PICU (2022 to 2024), tocilizumab was used as an adjuvant therapy for pediatric refractory septic shock and reported significantly lower mortality rates compared with patients treated without tocilizumab in the earlier period (2018 to 2021).

A previous study reported that mortality among patients with pediatric septic shock and cancer ranged from 23% to 60% [1,21]. The median PRISM III score was 16 in the malignant group, which predicted a mortality rate of approximately 45% [22]. In our cohort, early tocilizumab administration for pediatric septic shock with hematological malignancy seemed to have a survival benefit and decreased shock duration. They presented with excessively high IL-6 levels (>1000 pg/mL), which significantly decreased after tocilizumab administration. The median shock duration was approximately 82 h, which was better than that reported in a previous study that reported that children with malignancies and septic shock experienced a median shock duration of 96 h [23]. The mortality rate at 28 days was 27.2%, which was better than previous studies and our previous hematologic patients (61.5%) [1,21,22].

Other patients with no history of hematological malignancy also received early tocilizumab for septic shock, and IL-6 levels also dropped after tocilizumab administration. The mortality rate at 28 days was zero in this group. The mortality rate among patients without hematological malignancy who did not receive tocilizumab in the earlier period (2018 to 2021) was 50% (10/20). Because IL-6/sIL-6R is involved in inducing thrombosis, vascular damage, myocardial impairment, and multiorgan dysfunction, the humanized anti-IL-6 receptor antibody tocilizumab will block the IL-6-mediated signaling pathway by inhibiting IL-6 binding to both the soluble and transmembrane forms of IL-6Rs. The deletion of IL-6 activity suppresses the evolution of multiorgan damage [11]. Our study may indirectly confirm the theory that tocilizumab may be beneficial and can shorten the duration of shock.

There may be several causes of septic shock in children with an underlying disease of hematological malignancy that presents with excessively high IL-6 levels. These children with malignancy were immunosuppressed, particularly the adaptive immune system; therefore, innate immunity, such as monocytes and macrophages, will over-activate and produce excessive IL-6 after sepsis [11]. Another possible reason is that the original malignancy had not been cured and remained in an inflammatory state. Therefore, in patients with malignancy, it is crucial to monitor the inflammatory status after sepsis and consider using molecular-targeted therapies, such as tocilizumab, for elevated IL-6 levels. Further randomized clinical trials are required to identify the appropriate population and timing of the administration of IL-6 receptor antagonists for patients with sepsis.

## 5. Limitations of the Study

This study has several limitations. First, the sample size was small and retrospectively conducted at a single center, and therefore, there were risks of missing data and information bias. However, similar results have been reported in adult and animal studies. Second, the study was conducted over a relatively long period (7 years) during which the therapeutic strategy may have evolved, such as the choice of parameters for resuscitation end-points or the available therapeutic armamentarium were different between these groups, which may have impacted the outcomes. However, during the study period, we followed the guidelines of early goal-directed therapy in the treatment of septic shock, which may have reduced the risk of bias. Third, three patients underwent mechanical ventilation, one of whom had a tracheostomy and received tocilizumab during the later study period (2022–2024). We did not analyze the impact of a protective ventilation strategy on plasma cytokine levels in the current study [24]. Additionally, we did not conduct a separate analysis of outcomes in tracheostomized patients with septic shock who received tocilizumab. Previous studies demonstrated survival benefits in tracheostomy patients [25,26]. Future studies are warranted to explore this subset of patients in greater detail.

## 6. Conclusions

The study found that tocilizumab may potentially help mitigate systemic inflammation in septic children with excessive IL-6 levels and may improve survival rates in children with septic shock. Further high-quality randomized controlled trials are warranted to evaluate the potential risks and benefits of tocilizumab for pediatric septic shock.

## Figures and Tables

**Table 1:** Demographics and initial parameters between patients with refractory septic shock treated with and without tocilizumab.

Variables	Patients Treated Without Tocilizumab (n = 33)	Patients Treated with Tocilizumab (n = 21)	*p*-Value
Age (years)	12.2 ± 4.3	13.6 ± 4.8	0.78
Sex (male), n (%)	16 (48.5)	9 (42.5)	0.68
Weight (kg)	35.6 ± 14.8	42 ± 15.2	0.65
Underlying, n (%)	23 (63.6)	17 (80)	0.35
PRISM III score	15.8 ± 4.3	13.8 ± 3.9	0.52
Site of infection, n (%)			0.25
Central nervous system	5 (15.1)	2 (10.5)	
Blood stream	13 (39.4)	11 (52.3)	
Respiratory	7 (21.2)	4 (21)	
Urologic	1 (3)	1 (5.2)	
Abdominal	1 (3)	2 (10.5)	
Others	6 (18.2)	1 (5.2)	
Culture positive, n (%)	24 (72.7)	13 (68.4)	
Pathogen, n (%)			0.32
Gram-positive	7 (21.2)	7 (36.8)	
Gram-negative	13 (39.4)	10 (50)	
Fungus	2 (6)	0	
Virus	2 (6)	2 (10.5)	
Unknown	9 (27.3)	2 (9.5)	
Cardiac characteristics			
Vasoactive-inotropic scores	44.6 ± 13.8	40.3 ± 11.2	0.71
Heart rate (beats/min)	132.3 ± 28.9	112.5 ± 22.3	0.68
Mean arterial pressure (mm Hg)	70 ± 20.5	68.3 ± 19.5	0.75
Outcomes			
ICU stay (days)	17.6 ± 12.5	7.3 ± 3.2	0.02 *
Mortality, n (%)	18 (54.5)	3 (14.2)	0.03 *

Abbreviations: PRISM, Pediatric Risk of Mortality; ICU, intensive care unit. * Statistical significance was set at *p* < 0.05.

**Table 2:** Comparisons in non-hematologic group.

Variables	Patients Treated Without Tocilizumab (n = 20)	Patients Treated with Tocilizumab (n = 10)	*p*-Value
Age (years)	10.8 ± 4.4	9.3 ± 6.8	0.49
Sex (male), n (%)	11 (55)	3 (30)	0.19
Weight (kg)	32.6 ± 13.8	30.1 ± 20.6	0.48
Underlying, n (%)	10 (45)	6 (60)	0.54
PRISM III score	13.6 ± 3.5	12.6 ± 2	0.11
Site of infection, n (%)			0.83
Central nervous system	5 (25)	2 (20)	
Blood stream	6 (30)	4 (40)	
Respiratory	5 (25)	3 (30)	
Urologic	0	1 (10)	
Abdominal	0	2 (20)	
Others	4 (20)	1 (10)	
Culture positive, n (%)	12 (60)	8 (80)	
Pathogen, n (%)			0.12
Gram-positive	4 (20)	5 (50)	
Gram-negative	6 (30)	1 (10)	
Fungus	0	0	
Virus	2 (10)	2 (20)	
Unknown	8 (40)	2 (20)	
Laboratory examination, median (IQR)			
WBC (u/L)	13,800 (8700–17,200)	12,300 (9400–16,500)	0.52
Hemoglobin (g/dL)	11.2 (9.5–13.5)	12 (10.5–12.1)	0.38
Platelet (∗10^3^)	175 (145–243)	212 (165–263)	0.46
Creatinine (mg/dL)	0.54 (0.22–0.78)	0.67 (0.27–0.83)	0.39
GOT (U/L)	45 (22–61)	36 (25–51)	0.42
GPT (U/L)	35 (20–75)	24 (17–63)	0.44
Cardiac characteristics			
Vasoactive-inotropic scores	43.3 ± 10.8	38.3 ± 9.2	0.43
Heart rate (beats/min)	128.3 ± 25.9	109.5 ± 20.3	0.24
Mean arterial pressure (mm Hg)	72 ± 18.3	72.3 ± 18.5	0.82
Outcomes			
ICU stay (days)	15.7 ± 9.1	5.7 ± 4.1	0.004 *
Mortality, n (%)	10 (50)	0	0.006 *

Abbreviations: PRISM, Pediatric Risk of Mortality; WBC, white blood cell; GOT, glutamic-oxaloacetic transaminase; GPT, glutamic-pyruvic transaminase; ICU, intensive care unit. * Statistical significance was set at *p* < 0.05.

**Table 3:** Comparisons in hematologic group.

Variables	Patients Treated Without Tocilizumab (n = 13)	Patients Treated With Tocilizumab (n = 11)	*p*-Value
Age (years)	14.3 ± 3.3	15.5 ± 6.6	0.23
Sex (male), n (%)	5 (38.4)	6 (54.5)	0.43
Weight (kg)	40.4 ± 15.7	50.2 ± 24.3	0.25
Underlying, n (%)			
PRISM III score	18.3 ± 3.1	16.2 ± 2.8	0.14
Site of infection, n (%)			0.71
Blood stream	7 (53.8)	7 (63.6)	
Respiratory	2 (15.3)	1 (9)	
Urologic	1 (7.6)	0	
Abdominal	1 (7.6)	0	
Others	2 (15.3)	0	
Culture positive, n (%)	12 (92.3)	11 (100)	
Pathogen, n (%)			0.39
Gram-positive	3 (23)	2 (18.1)	
Gram-negative	7 (53.8)	9 (81.8)	
Fungus	2 (15.3)	0	
Unknown	1 (7.6)	0	
Laboratory examination, median (IQR)			
WBC (u/L)	310 (120–870)	200 (107–750)	0.47
Hemoglobin (g/dL)	10.8 (7.5–11.2)	10.1 (8.6–10.4)	0.45
Platelet (∗10^3^)	18 (9–32)	11 (7–25)	0.58
Creatinine (mg/dL)	0.61 (0.47–1.24)	0.57 (0.49–1.17)	0.48
GOT (U/L)	41 (24–71)	34 (22–62)	0.41
GPT (U/L)	41 (16–131)	45 (14–140)	0.39
Cardiac characteristics			
Vasoactive-inotropic scores	48.2 ± 15.3	44.3 ± 13.2	0.74
Heart rate (beats/min)	136.3 ± 29.9	115.3 ± 24.3	0.64
Mean arterial pressure (mm Hg)	66 ± 18.5	67.3 ± 17.5	0.71
Outcomes			
ICU stay (days)	20.3 ± 17.2	8.7 ± 6.2	0.01 *
Mortality, n (%)	8 (61.5)	3 (27.2)	0.09

Abbreviations: PRISM, Pediatric Risk of Mortality; WBC, white blood cell; GOT, glutamic-oxaloacetic transaminase; GPT, glutamic-pyruvic transaminase; ICU, intensive care unit. * Statistical significance was set at *p* < 0.05.

## Data Availability

The datasets used and analyzed during the current study are available from the corresponding author upon reasonable request.
